# Examining the predictive validity of alcohol‐seeking following punishment‐imposed abstinence in mice

**DOI:** 10.1111/acer.70057

**Published:** 2025-05-27

**Authors:** Linh Tran, Maria Kuznetsova, Elizabeth E. Manning, Erin J. Campbell

**Affiliations:** ^1^ Faculty of Health and Medicine, School of Biomedical Sciences and Pharmacy University of Newcastle Callaghan New South Wales Australia; ^2^ Brain Neuromodulation Research Program Hunter Medical Research Institute New Lambton Heights New South Wales Australia

**Keywords:** c57 mice, context, foot shock, punishment, relapse

## Abstract

**Background:**

A defining feature of alcohol use disorder that has captured the attention of fundamental researchers is “persistent use despite negative consequences.” The last two decades have seen the preclinical field adopt the use of punishment to model the adverse consequences associated with alcohol use. However, existing research has focused on rats as the model of choice and alcohol consumption as the prevailing outcome measure. Additionally, the predictive validity of these models, that is, testing currently approved FDA treatments, is yet to be realized.

**Methods:**

Here, we examined punishment‐imposed abstinence in mice using foot shock and measured reinstatement of alcohol‐seeking following exposure to alcohol‐associated cues and environmental contexts.

**Results:**

We showed that mice voluntarily abstain from alcohol use when it is paired with a foot shock. Alcohol‐associated cues and environmental contexts produced reinstatement of alcohol‐seeking behavior. Finally, the predictive validity of our model was tested using naltrexone and varenicline, two medications to treat alcohol use disorder. Both naltrexone and varenicline reduced reinstatement of alcohol‐seeking in male and female mice.

**Conclusions:**

Together, these data suggest that mice can display reinstatement of alcohol‐seeking behavior following voluntary abstinence, and this model could be used to identify new medications for relapse prevention induced by environmental cues and contexts.

## Graphical abstract

Animal models of alcohol use disorder are critical to understand the neurochemical mechanisms underlying this disorder to inform targeted medication development. However, quite often the validity of commonly used animal models remains untested. Here, we optimized a highly used model of abstinence in mice and demonstrated the predictive validity of the model using naltrexone and varenicline, medications currently used in people with alcohol use disorder. Our data highlight the validity of this model to identify new, targeted treatments for the reinstatement of alcohol use.

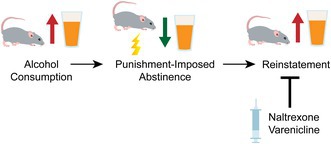

## INTRODUCTION

The unhealthy consumption of alcohol is widespread, creating huge public health concerns, and increasing the risk of developing alcohol use disorder (Australian Institute of Health and Welfare, 2024; World Health Organization, 2018). Alcohol use disorder is a chronic condition with relapse continuing to be a serious obstacle for treatment (Miller et al., 2001). Importantly, environmental factors such as alcohol‐associated cues and contexts often provoke relapse in humans and relapse‐like behavior in rodents (Liu & Weiss, 2002; Niaura et al., 1988). The last several decades have seen the use of the reinstatement model in rodents to examine the return to harmful drinking behaviors after a period of abstinence (Shaham et al., 2003). In this model, rodents are trained in an operant setting to press a lever for an alcohol reward. A lever press is usually associated with discrete cues or environmental contexts. Following this, rodents typically undergo a period of experimenter‐imposed extinction where the rodent is forced into an “alcohol free” environment in the absence of alcohol‐associated cues (Bossert et al., 2013; Prasad & McNally, 2016; Tsiang & Janak, 2006). After a period of forced abstinence, rodents are examined for alcohol‐seeking behavior following reexposure to the cues or contexts initially associated with alcohol use.

One key limitation of extinction–reinstatement models is that abstinence from alcohol is usually enforced by the experimenter, which hampers translation. One adaptation of this model has incorporated the use of punishment to model the negative consequences of substance use that often lead to voluntary abstinence in humans (Cooper et al., 2007; Marchant et al., 2013; Panlilio et al., 2003). Electric foot shock is often used as a negative consequence in these reinstatement paradigms due to its flexibility in intensity, timing of delivery, and frequency (Maddern, Walker, et al., 2024). In this adapted model, a lever press still results in an alcohol reward but is also associated with foot shock. Rodents learn to associate different cues or environmental contexts with foot shock. This models self‐imposed abstinence in people with alcohol use disorder, which is typically voluntary due to the negative consequences of alcohol use (with exceptions such as incarceration). This adapted model has transformed the field and has overcome a limitation of traditional reinstatement models, where abstinence was controlled by the experimenter. However, most studies utilizing the adapted voluntary abstinence model with foot shock have focused on rats as the model of choice (Fredriksson et al., 2021). While important, mechanistic research of these behaviors in mice can be facilitated by the wide availability and abundance of transgenic mouse lines in comparison with transgenic rat lines.

Here, we aimed to optimize a mouse model of alcohol‐seeking following punishment‐imposed abstinence. To do this, we adapted the context‐induced reinstatement model where we trained mice to self‐administer alcohol in an environment associated with several discrete and contextual cues (Context A; Crombag & Shaham, 2002; Tsiang & Janak, 2006). We saw reliably high alcohol consumption during self‐administration training in both male and female mice (~1 g/kg/30 min). We then punished the operant self‐administration response in a different environmental context where a lever press for alcohol was paired with 0.2–0.3 mA foot shock (Context B). Here, both male and female mice reduced their operant responding for alcohol when it was paired with a negative consequence. We then observed alcohol‐seeking behavior in the original alcohol‐associated environment (Context A) but not the punishment‐associated environment (Context B). Thus, we were able to replicate alcohol reinstatement findings from rats in mice, using the punishment‐imposed abstinence model.

Following on from this, we sought to further understand the validity of this model by testing the effect of currently approved or effective medications. In the alcohol use disorder field, naltrexone is a nonselective opioid receptor antagonist and is currently FDA‐approved to reduce craving in individuals with alcohol use disorder (Campbell et al., 2018; Maisel et al., 2013). Another medication that has shown promise at reducing alcohol craving in humans is varenicline, an α4β2 nicotinic acetylcholine receptor partial agonist (Gandhi et al., 2020; Litten et al., 2013; McKee et al., 2009). Thus, to further understand the validity of this punishment‐imposed abstinence model in mice, we examined the predictive validity by testing the effect of naltrexone and varenicline on alcohol‐seeking behavior. Naltrexone and varenicline reduced the reinstatement of alcohol‐seeking behavior in both sexes. Together, these data enhance the validity of the punishment‐imposed abstinence model by replicating findings from rats in mice and by testing current human medications in this model.

## MATERIALS AND METHODS

### Ethics statement

All experiments were performed in accordance with the National Health and Medical Research Council (NHMRC) Australian Code for the Care and Use of Animals for Scientific Purposes (2013) and approved by The University of Newcastle Animal Care and Ethics Committee.

### Animals

A total of 36 male and 34 female C57BL/6JAusB mice (~8 weeks old) were obtained from Australian BioResources, New South Wales. One male and four female mice were excluded because their alcohol consumption was low throughout Context A self‐administration training (<0.3 g/kg/session). Mice were group housed during experiments (2–5 mice per cage) with standard bedding and enrichment. Food (Gordon's Speciality Feed) and water were available ad libitum, and all mice were maintained on a reverse 12‐h light/dark cycle (0700 lights off).

### Apparatus

Standard operant chambers (Med Associates) enclosed in sound‐attenuating chambers were used for self‐administration. Each chamber was equipped with two retractable levers on either side of a central magazine receptacle. An active lever press resulted in the delivery of 10% ethanol (13 μL/delivery) into the receptacle via a 19‐gauge needle connected to a 3 mL syringe, controlled by a PHM‐200 syringe pump (Med Associates). An inactive lever press had no programmed consequence. Grid floors were connected to shockers (ENV‐414, Med Associates). Contexts A and B were manipulated in a similar way to our previous studies (Campbell et al., 2019, 2021): illumination level (cue/no cue light), background (stripes/none), olfaction (vanilla essence/none), and background noise (white noise off/on). The context used during different experimental phases was counterbalanced across mice, and no effects of context on alcohol consumption at baseline were observed.

### Experiment 1: Alcohol‐seeking after punishment‐imposed abstinence in mice

**FIGURE 1:**
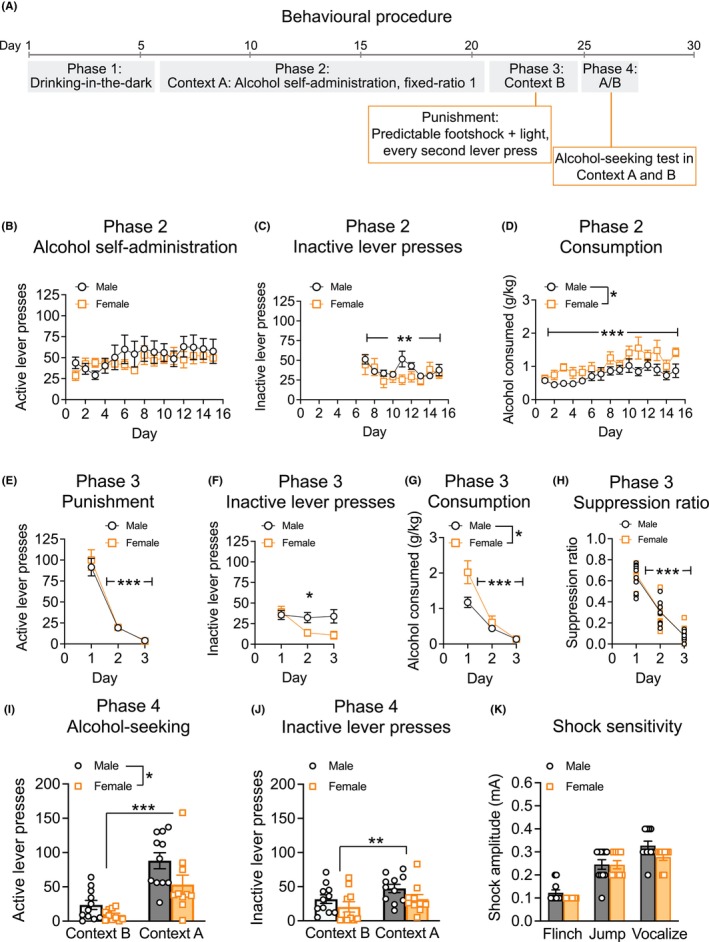
Alcohol‐seeking after punishment‐imposed abstinence in mice. (A) Outline of the behavioral procedure for Experiment 1. (B) Both sexes reliably pressed the active lever for alcohol under a fixed‐ratio 1 schedule of reinforcement in Context A. (C) There was a reduction in the number of inactive lever presses across Day in Context A. (D) Mice consumed more alcohol (g/kg) across Day during Context A self‐administration and female mice consumed more alcohol compared to male mice. (E) Predictable foot shock reduced lever responding for alcohol in Context B. The foot shock punishment ranged from 0 mA on Day 1, to 0.2 mA on Day 2 and 0.3 mA on Day 3. (F) Females reduced inactive lever presses across Day during punishment sessions but there was no change in inactive lever presses for male mice. (G) Alcohol consumption decreased across punishment days for both males and females, but females consumed more alcohol (g/kg) compared to males during Context B punishment. (H) Suppression ratio during Context B, punishment, reduced across Day. (I) Greater alcohol‐seeking behavior was observed in Context A compared to Context B. Male mice had a greater number of active lever presses compared to female mice. (J) There were more inactive lever presses in Context A compared to Context B. (K) There were no sex differences in shock sensitivity when mice were observed for their behavioral response (flinch, jump, vocalize) to increasing amplitudes of foot shock. Data presented as mean ± standard error mean. *p < 0.05; **p < 0.01; ***p < 0.001.

A total of 11 male mice and 10 female mice were used in this study. The behavioral procedure was adapted from our previously published work in rats (Figure 1A) (Campbell et al., 2019).

### Behavioral procedure (four phases)

#### Phase 1: Drinking‐in‐the‐dark alcohol intake

A drinking‐in‐the‐dark procedure was implemented where water bottles were replaced with bottles of 10% ethanol for 2 h beginning 3 h into the dark cycle (i.e., 10 am) (Rhodes et al., 2005; Thiele & Navarro, 2014). This procedure was adopted from Rhodes et al. (2005) to preexpose mice to alcohol in a stress‐free environment (i.e., the home cage). Alcohol was given to mice at 10 weeks of age on alternating days of the week for six sessions. Alcohol bottles were prepared in tap water from 100% (v/v) ethanol and weighed before and after the session to measure consumption. Average spillage was accounted for using a “spill” bottle with 10% ethanol in an empty cage during the session. Total alcohol consumption in grams was calculated for each session, using the weight difference between the beginning and end of the session, minus spillage, multiplied by 0.986 (density of 10% ethanol) and divided by the weight of mice in the cage. It should be noted that mice were group housed throughout experimentation and thus alcohol consumption during the drinking‐in‐the‐dark experimental phase was averaged for each mouse.

#### Phase 2: Operant self‐administration: Context A

Mice underwent daily (weekday) operant sessions for 30 min, under a fixed‐ratio (FR‐1) schedule of reinforcement. Under this schedule, one active lever press resulted in the delivery of 13 μL of 10% ethanol paired with a two‐second tone‐cue. This was followed by a three‐second timeout period where lever presses were recorded but not reinforced. To facilitate operant acquisition, the inactive lever was absent during the first six sessions and introduced at Session 7. A total of 15 sessions were conducted in Context A. For accurate alcohol consumption measurements at the end of the operant session, residual solution in the central receptacle was withdrawn with a 1 mL syringe and recorded. Context A training was conducted between 9 am and 10:30 am.

#### Phase 3: Punishment‐imposed voluntary abstinence: Context B

Following Phase 2, mice were trained to self‐administer alcohol in an alternate context (Context B) under the same FR‐1 administration schedule described above. However, every second reinforced active lever press resulted in the delivery of a 0.5 s foot shock. Thus, punished active lever responses resulted in a foot shock, 2 s house light illumination, alcohol delivery, and 2 s tone. Inactive lever presses had no programmed consequence. Mice were punished in Context B for at least 3 days. Day 1 in Context B had no delivery of foot shock and served as a habituation day to the change in environment. The following day, every second lever press resulted in the delivery of a 0.2‐mA foot shock. The third punishment day resulted in the delivery of a 0.3‐mA foot shock. An extra punishment day was conducted at 0.3 mA for any mice that had greater than 50 active lever presses on Day 2.

#### Phase 4: Context‐induced alcohol‐seeking

Mice underwent 2 days of alcohol‐seeking tests the day after Context B punishment‐imposed voluntary abstinence. Here, mice were examined for alcohol‐seeking behavior in both Context A and Context B. The order of testing of the two contexts was counterbalanced. Seeking tests followed an FR‐1 schedule of reinforcement where an active lever press resulted in the delivery of the 2 s tone; however, no alcohol or foot shock was delivered.

#### Shock sensitivity

Foot shock sensitivity was examined 7 days after the alcohol‐seeking test in a subset of mice from Experiment 1 (11 males, 9 females). Mice were placed in a novel operant chamber in a sound‐attenuating cubicle (Med Associates), with no access to levers or magazines. The grid floor was connected to a shocker. Mice were placed into the operant chamber and given 3 min to habituate to the chamber. Following this, a foot shock was delivered every minute starting at 0.1 mA intensity and increasing in intensity by 0.1 mA each minute. The behavioral response to the shock was recorded and scored by two observers blinded to sex. A flinch was operationalized as an observable reaction to the shock (e.g., lifting or shaking their paws), a jump was defined as any jumping, prancing, or running following shock, and a vocalization was operationalized as an audible squeak following shock (Kim et al., 1991; Sneddon et al., 2022). The experiment ended when an audible vocalization was heard. The maximum shock intensity delivered was 0.5 mA.

### Experiment 2: Effects of naltrexone and varenicline on alcohol‐seeking in mice

#### Experiment 2a: Alcohol‐seeking behavior following acute naltrexone administration

**FIGURE 2:**
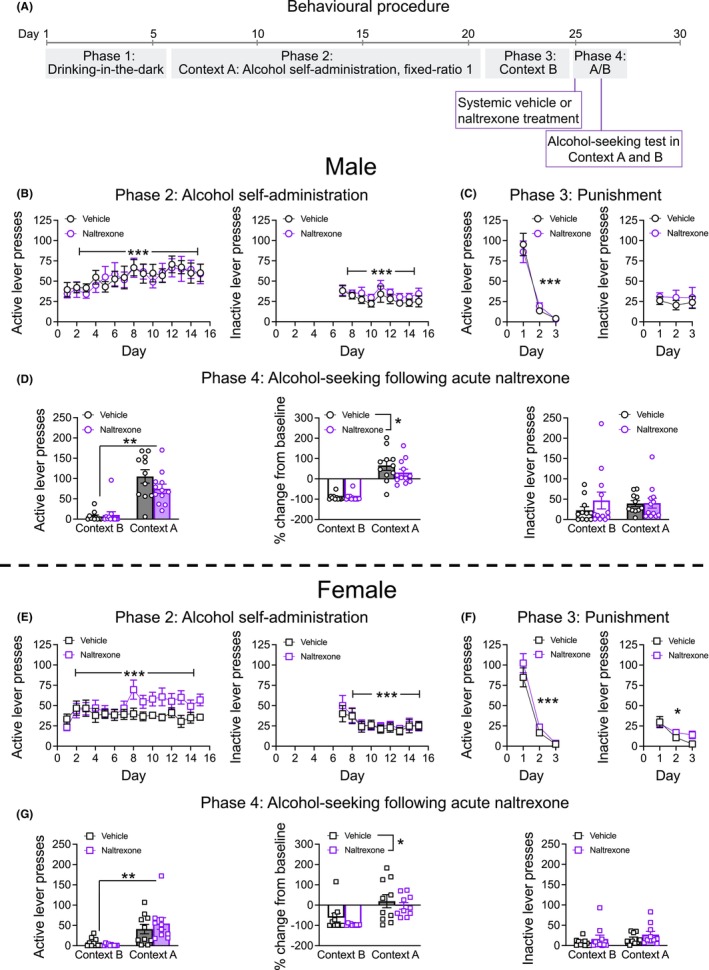
Experiment 2a: Alcohol‐seeking behavior following acute naltrexone administration. (A) Outline of the behavioral procedure for Experiment 2a. (B) Male mice increased active lever responding for alcohol across Day under a fixed‐ratio 1 schedule of reinforcement in Context A (left). Inactive lever responding reduced across Day in Context A (right). (C) There was a reduction in the number of active lever presses across Day in Context B for male mice (left). There was no change in the number of inactive lever presses in Context B for male mice (right). (D) Following acute naltrexone administration (1 mg/kg, subcutaneous), male mice had increased alcohol‐seeking behavior in Context A versus Context B (left). When accounting for baseline responding, naltrexone reduced the number of active lever presses compared to vehicle controls (middle). There was no effect of naltrexone on the number of inactive lever presses in male mice during the alcohol‐seeking test (right). (E) Female mice increased active lever responding for alcohol across Day under a fixed‐ratio 1 schedule of reinforcement in Context A (left). Inactive lever responding reduced across Day in Context A (right). (F) There was a reduction in the number of active lever presses across Day in Context B for female mice (left). There was also a reduction in the number of inactive lever presses in Context B for female mice (right). (G) Following acute naltrexone administration (1 mg/kg, subcutaneous), female mice had increased alcohol‐seeking behavior in Context A versus Context B (left). When accounting for baseline active lever pressing, naltrexone reduced the number of active lever presses compared to vehicle controls (middle). There was no effect of naltrexone on the number of inactive lever presses in female mice during the alcohol‐seeking test (right). Data presented as mean ± standard error mean. *p < 0.05; **p < 0.01; ***p < 0.001.

A total of 23 male mice and 20 female mice were used in this study. 11 male mice and 10 female mice from Experiment 1 were retrained in Context A for three sessions and repunished in Context B for two sessions, which was followed by acute naltrexone administration for the alcohol‐seeking test. A further 12 male mice and 10 female mice were obtained from Australian BioResources, New South Wales, and underwent Phases 1–4 as described in Experiment 1 above. Mice were habituated to subcutaneous (s.c) saline injections 15 min prior to 2 × Context A sessions and 1 × Context B session. On alcohol‐seeking test days, mice were randomly assigned to either the vehicle (0.9% sterile saline, 10 mL/kg, s.c) or naltrexone (1 mg/kg, Tocris, Lot number = 7A/287286, dissolved in saline and delivered at 10 mL/kg, s.c) group and given injections 15 min before the alcohol‐seeking test (Middaugh & Bandy, 2000; Williams & Broadbridge, 2009). The drug type assigned to each mouse remained the same for the two test days, with Context counterbalanced across test days. Refer to Figure 2A for behavioral procedure.

#### Experiment 2b: Alcohol‐seeking behavior following acute varenicline administration

**FIGURE 3:**
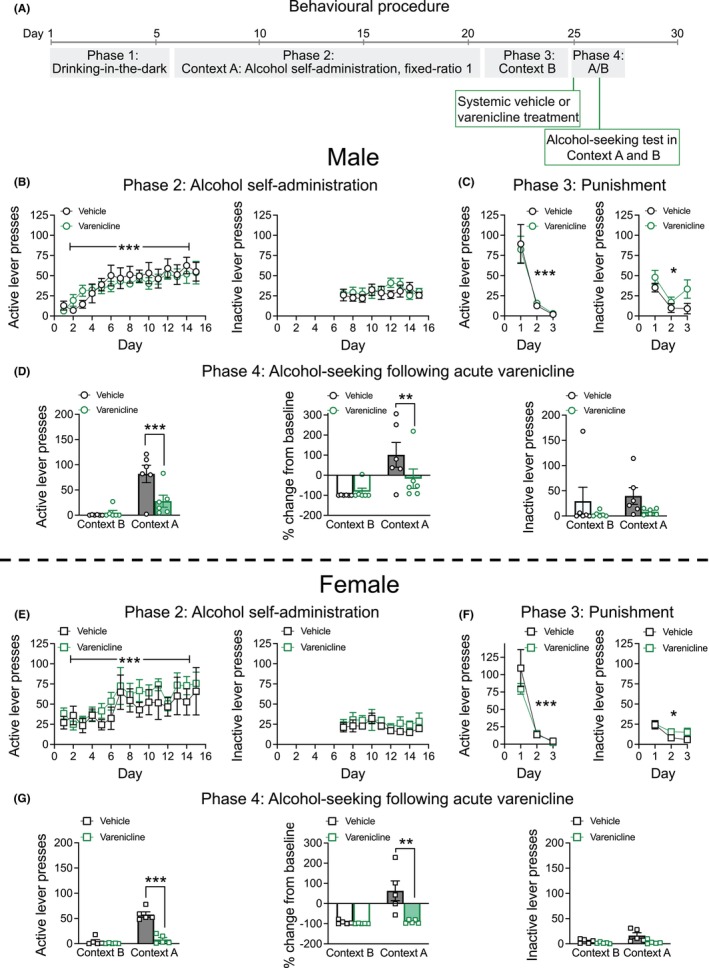
Experiment 2b: Alcohol‐seeking behavior following acute varenicline administration. (A) Outline of the behavioral procedure for Experiment 2b. (B) Male mice increased active lever responding for alcohol across Day under a fixed‐ratio 1 schedule of reinforcement in Context A (left). Inactive lever responding did not change across Day in Context A (right). (C) There was a reduction in the number of active lever presses across Day in Context B for male mice (left). There was also a reduction in the number of inactive lever presses in Context B for male mice (right). (D) Acute varenicline administration (3 mg/kg, subcutaneous), reduced alcohol‐seeking behavior in male mice in Context A (left). Additionally, when accounting for baseline responding, varenicline reduced the number of active lever presses compared to vehicle controls in Context A (middle). There was no effect of varenicline on the number of inactive lever presses in male mice during the alcohol‐seeking test (right). (E) Female mice increased active lever responding for alcohol across Day under a fixed‐ratio 1 schedule of reinforcement in Context A (left). There was no change in the number of inactive lever presses in Context A (right). (F) There was a reduction in the number of active lever presses across Day in Context B for female mice (left). There was also a reduction in the number of inactive lever presses in Context B for female mice (right). (G) Acute varenicline administration (3 mg/kg, subcutaneous) reduced alcohol‐seeking behavior in female mice in Context A (left). When accounting for baseline active lever pressing, varenicline reduced the number of active lever presses compared to vehicle controls in Context A (middle). There was no effect of varenicline on the number of inactive lever presses in female mice during the alcohol‐seeking test (right). Data presented as mean ± standard error mean. *p < 0.05; **p < 0.01; ***p < 0.001.

A total of 12 male mice and 10 female mice were obtained from Australian BioResources, New South Wales, for this study. Mice underwent Phases 1–4 as described in Experiment 1 above. Mice were habituated to subcutaneous (s.c) saline injections 20 min prior to 2 × Context A sessions and 1 × Context B session. On alcohol‐seeking test days, mice were randomly assigned to either the vehicle (0.9% sterile saline, 10 mL/kg, s.c) or varenicline (3 mg/kg, Sigma, Lot number = 0000147464, dissolved in saline and delivered at 10 mL/kg, s.c) group and given injections 20 min before the alcohol‐seeking test (Lacroix et al., 2017; Randall et al., 2015; Wouda et al., 2011). The drug type assigned to each mouse remained the same for both test days, with Context counterbalanced across test days. Refer to Figure 3A for the behavioral procedure.

### Statistical analysis

Statistical analyses were conducted using JASP V16.2. Data were analyzed separately for the four behavioral phases: drinking‐in‐the‐dark, Context A self‐administration, Context B punishment, and the alcohol‐seeking tests. For Experiment 1, drinking‐in‐the‐dark, self‐administration, and punishment data were analyzed using a repeated measures analysis of variance (ANOVA) examining a within‐subjects effect of Day and a between‐subjects effect of Sex (Male, Female). Alcohol consumption during operant sessions was calculated as (alcohol deliveries × 13 μL – remaining alcohol in receptacle at the end of the session)/(mouse weight in grams). The suppression ratio was calculated as (punishment alcohol deliveries)/(punishment alcohol deliveries + average alcohol deliveries of the last three Context A sessions). A suppression ratio score >0.5 represents an increase in lever responding during punishment. A suppression ratio score <0.5 represents a reduction in lever responding during punishment. A Pearson's correlation assessed the relationship between alcohol consumption and suppression ratio. ANOVA was used to analyze the alcohol‐seeking test in Experiment 1; the within‐subjects factor was Context (Context A, Context B) and the between‐subjects factor was Sex. Test Context Order was also included as a covariate for results from Phase 4. For Experiment 2, drinking‐in‐the‐dark, self‐administration, and punishment data were analyzed using a repeated measures analysis of variance (ANOVA) examining a within‐subjects effect of Day and a between‐subjects effect of Sex (Male, Female) and Drug Group Allocation (Vehicle, Naltrexone or Varenicline). For Experiment 2 alcohol‐seeking tests, the within‐subjects factor was Context, and the between‐subjects factors were Sex and Drug treatment (Vehicle, Naltrexone, or Varenicline). The percentage change from baseline was calculated as ((alcohol‐seeking test active lever presses) – (average active lever presses of the last 5 Context A sessions))/(average active lever presses of the last 5 Context A sessions) * 100. Shock sensitivity was analyzed as a repeated measures ANOVA with the within‐subjects factor of Response (Flinch, Jump, and Vocalize). Tests of sphericity were assessed for all within‐subjects ANOVAs; if sphericity was violated, Greenhouse–Geisser corrections were reported. Significant interaction effects (*p* < 0.05) were followed up with Tukey's post hoc comparisons.

## RESULTS

### Experiment 1: Alcohol‐seeking after punishment‐imposed abstinence in mice

#### Drinking‐in‐the‐dark and Context A alcohol self‐administration

Male and female C57BL/6J mice were exposed to 10% (v/v) alcohol in their home cage prior to operant training. During drinking‐in‐the‐dark home cage sessions, there were day‐dependent effects of sex (Figure S1A; Sex × Day interaction *F*_2.2,42_ = 3.776, *p* = 0.027). Post hoc tests revealed that females had reduced consumption on Day 5 (*p* < 0.05), whereas on other days, they were marginally higher, although this did not reach statistical significance. When assessing active lever presses during Phase 2, Context A self‐administration, there was a gradual increase in active lever presses across days as expected (Figure 1B; main effect of Day *F*_3.6,67.6_ = 2.556, *p* = 0.053; Sex × Day interaction *p* > 0.05). During Context A self‐administration, there was a reduction in inactive lever presses across days in both male and female mice (Figure 1C; Sex × Day interaction *F*_2.3,43.3_ = 3.784, *p* = 0.026; main effect of Day *F*_2.3,43.3_ = 5.702, *p* = 0.005). Mice reliably consumed high quantities of alcohol in Context A (Figure 1D; ~1 g/kg/30 min). Consumption increased throughout Context A training as expected and female mice consumed more alcohol compared to male mice (Figure 1D; main effect of Sex *F*_1,19_ = 6.971, *p* = 0.016; main effect of Day *F*_4.4,84_ = 5.675, *p* < 0.001; Sex × Day interaction *p* > 0.05).

#### Context B punishment

Both male and female mice demonstrated punishment‐imposed suppression of alcohol‐reinforced lever pressing in Context B using contingent foot shock (Figure 1E; main effect of Day *F*_1.1,20.8_ = 122.219, *p* < 0.001; Sex × Day interaction *p* > 0.05). For Context B inactive lever presses, there were day‐dependent effects of sex (Figure 1F; Sex × Punishment day interaction *F*_1.4,25.8_ = 5.511, *p* = 0.019) and post hoc tests revealed that this was driven by a reduction in inactive lever responding in female mice across punishment days (*p*'s < 0.05) but no change in inactive lever responding for male mice across subsequent punishment days (*p*'s > 0.05). Females consumed more alcohol compared to males during Context B (Figure 1G; main effect of Sex *F*_1,19_ = 6.816, *p* = 0.017) but overall consumption during Context B decreased across days (Figure 1G; main effect of Day *F*_1.2,23.3_ = 42.265, *p* < 0.001; Sex × Punishment Day interaction *p* > 0.05). When we analyzed punishment suppression ratio, accounting for individual differences in baseline self‐administration, responding for alcohol was suppressed across punishment days (Figure 1H; main effect of Day *F*_2,38_ = 305.015, *p* < 0.001; Sex × Day interaction *p* > 0.05). Alcohol consumption on the final day of operant self‐administration had a moderate, negative correlation with suppression ratio the first day shock was present (*r* = −0.513, *p* = 0.017).

#### Alcohol‐seeking behavior following punishment‐imposed abstinence

Both male and female mice had increased alcohol‐seeking behavior in Context A versus Context B (Figure 1I; main effect of Context *F*_1,19_ = 38.135, *p* < 0.001). Additionally, male mice pressed the active lever more compared to female mice during the alcohol‐seeking reinstatement test irrespective of context (Figure 1I; main effect of Sex *F*_1,19_ = 5.488, *p* = 0.030; Sex × Context interaction *p* > 0.05). These effects appeared consistent regardless of the order of the Context the mice were first tested, and Test Context Order was not a significant covariate in the final alcohol‐seeking test (Figure S2; *p* > 0.05). All mice pressed the inactive lever more during Context A versus Context B (Figure 1J; main effect of Context *F*_1,19_ = 10.891, *p* = 0.004). Finally, there were no significant sex differences across the three behavioral responses measured during the shock sensitivity task (Figure 1K; Sex × Response interaction *F*_2,36_ = 1.296, *p* = 0.286).

### Experiment 2a: Alcohol‐seeking behavior following acute naltrexone administration

#### Drinking‐in‐the‐dark and Context A alcohol self‐administration

Similar to Experiment 1, there were day‐dependent effects of sex on alcohol consumption during the drinking‐in‐the‐dark phase (Figures S1B,C; Sex × Day interaction *F*_2.6,101_ = 4.909, *p* = 0.005). Post hoc tests revealed that males had increased consumption on Day 4 (*p* < 0.05), whereas on other days, they were marginally lower, although this did not reach statistical significance. Importantly, there was no significant effect of Drug Group allocation on the amount of alcohol consumed during the drinking‐in‐the‐dark phase (main effect of Drug Group *p* > 0.05). When examining active lever presses during Phase 2, Context A self‐administration, there was an increase in lever responding across days for both male and female mice (Figure 2B,E; main effect of Day *F*_6.1,236.2_ = 4.394, *p* < 0.001; Sex × Day interaction *p* > 0.05). Importantly, there was no significant main effect of Drug Group allocation on Context A active lever presses (*p* > 0.05). Figure 2E appears to show that the naltrexone‐allocated female mice pressed the active lever more than the vehicle‐allocated females once the inactive lever was introduced. However, when analyzing active lever presses from Day 7 onwards, there was no significant main effect of Drug Group allocation (*p* > 0.05). For inactive lever presses during Context A, there was a reduction in responding across days (Figure 2B,E; Sex × Day interaction *F*_3.8,146.8_ = 3.321, *p* = 0.014; main effect of Day *F*_3.8,146.8_ = 9.213, *p* < 0.001). Importantly, there was no main effect of Drug allocation on inactive lever presses (*p* > 0.05). Similar to Experiment 1, mice consumed high quantities of alcohol in Context A (~1 g/kg/30 min, data not shown) with female mice consuming more alcohol compared to male mice (main effect of Sex, *F*_1,39_ = 5.935, *p* = 0.020).

#### Context B punishment

Similar to Experiment 1, both male and female mice demonstrated punishment‐imposed suppression of alcohol‐reinforced lever pressing in Context B with contingent foot shock (Figure 2C,F; main effect of Day *F*_1.1,41.7_ = 176.332, *p* < 0.001; Sex × Day interaction *p* > 0.05). Additionally, there was no significant effect of Drug Group allocation on the number of active lever presses during Context B (main effect of Drug Group *p* > 0.05). Similar to Experiment 1, there were day‐dependent effects of sex on the number of inactive lever presses (Figure 2C,F; Sex × Punishment Day interaction *F*_1.3,49.4_ = 4.594, *p* = 0.029), and post hoc tests revealed that there was a reduction in inactive lever responding in female mice across punishment days (*p*'s < 0.05) but no change in inactive lever responding for male mice across subsequent punishment days (*p*'s > 0.05). Alcohol consumption decreased across punishment day in Context B (data not shown, main effect of Day *F*_1.2,46.3_ = 77.919, *p* < 0.001) and females consumed more alcohol compared to male mice during Context B (data not shown, main effect of Sex *F*_1,39_ = 7.675, *p* = 0.009; Sex × Punishment Day interaction *p* > 0.05). For suppression ratio, responding for alcohol was suppressed across subsequent punishment days (Figure S3A; main effect of Day *F*_2,78_ = 524.834, *p* < 0.001; Sex × Day interaction *p* > 0.05).

#### Alcohol‐seeking behavior following acute naltrexone administration

There was a significant Sex × Context × Drug treatment interaction on the number of active lever presses following acute naltrexone administration (Figure 2D,G; *F*_1,39_ = 4.325, *p* = 0.044). Test Context Order was not a significant covariate in the final alcohol‐seeking test and thus had no significant influence on active lever presses (*p* > 0.05). Two‐way interactions revealed males had a greater number of active lever presses in Context A compared to females (Figure 2D,G; Context × Sex interaction *F*_1,39_ = 8.939, *p* < 0.01; Tukey's post hoc *p* < 0.001), but there were no differences in the number of active lever presses between males and females in Context B (*p* > 0.05). Mice also pressed the active lever more during Context A versus Context B (Figure 2D,G; main effect of Context *F*_1,39_ = 91.991, *p* < 0.01). Naltrexone did not influence the number of active lever presses during the alcohol‐seeking reinstatement test (Context × Drug interaction *p* > 0.05). However, to account for individual differences in lever pressing, the percentage change from baseline was calculated. Here, naltrexone reduced lever responding in both male and female mice (Figure 2D,G; main effect of Drug *F*_1,39_ = 4.096, *p* = 0.050; all interaction effects *p* > 0.05). There was no significant Sex × Context × Drug interaction on the number of inactive lever presses (Figure 2D,G; *p* > 0.05).

### Experiment 2b: Alcohol‐seeking behavior following acute varenicline administration

#### Drinking‐in‐the‐dark and Context A alcohol self‐administration

Similar to Experiment 1, there were day‐dependent effects of sex on alcohol consumption during Phase 1: Drinking‐in‐the‐dark (Figures S1D,E; Sex × Day interaction *F*_1.7,31_ = 6.233, *p* = 0.007). Post hoc tests revealed that females increased consumption across day (*p*'s < 0.05) but males did not (*p*'s > 0.05). Importantly, there was no significant effect of varenicline Drug Group allocation on the amount of alcohol consumed during the drinking‐in‐the‐dark phase (main effect of Drug Group >0.05). During Phase 2, Context A self‐administration, active lever pressing for alcohol increased across day for both male and female mice (Figure 3B,E; main effect of Day *F*_4.8,85.7_ = 10.703, *p* < 0.001, Sex × Day interaction *p* > 0.05). Importantly, there was no significant main effect of varenicline Drug Group allocation on Context A active lever presses (main effect of Drug Group *p* > 0.05). There were day‐dependent effects of sex on inactive lever pressing during Context A (Figure 3B,E; Sex × Day interaction *F*_8,144_ = 2.198, *p* = 0.031), but post hoc tests revealed no significant differences across groups (*p*'s > 0.05). Importantly, there was no main effect of Drug allocation on inactive lever presses (*p* > 0.05). Similar to Experiments 1 and 2a, mice consumed high quantities of alcohol in Context A (~1 g/kg/30 min, data not shown) with consumption increasing across day (main effect of Day *F*_6.0,107.3_ = 6.446, *p* < 0.001).

#### Context B punishment

Similar to Experiments 1 and 2a above, both male and female mice demonstrated a reduction in the number of active lever presses in Context B with contingent foot shock (Figure 3C,F; main effect of Day *F*_1,18.2_ = 67.244, *p* < 0.001; Sex × Day interaction *p* > 0.05). Importantly, there was no significant effect of varenicline Drug Group allocation on the number of active lever presses during punishment (*p* > 0.05). There was a day‐dependent effect of sex on the number of inactive lever presses during Context B (Figure 3C,F; Sex × Punishment Day interaction *F*_2,36_ = 3.909, *p* = 0.029) and post hoc tests showed a reduction in inactive lever presses in male and female mice on day 1 of punishment versus days 2 and 3 (*p*'s < 0.05). Alcohol consumption decreased across days during Context B for both male and female mice (main effect of Day *F*_1.2,21.2_ = 35.5, *p* < 0.001, Sex × Punishment Day interaction *p* > 0.05). For suppression ratio, responding for alcohol was suppressed across subsequent punishment days (Figure S3B; main effect of Day *F*_2,36_ = 257.836, *p* < 0.001; Sex × Day interaction *F*_2,36_ = 14.478, *p* < 0.001). Post hoc tests revealed that female mice had greater suppression ratios on the first day of punishment compared to male mice (*p* < 0.05).

#### Alcohol‐seeking behavior following acute varenicline administration

There was no significant Sex × Context × Drug treatment interaction on the number of active lever presses following acute varenicline administration (*F*_1,18_ = 0.361, *p* = 0.555). Test Context Order was not a significant covariate in the final alcohol‐seeking test and had no significant influence on active lever presses (*p* > 0.05). Two‐way interactions revealed that varenicline significantly reduced active lever responding compared to vehicle in Context A (Figure 3D,G; Context × Drug treatment interaction *F*_1,18_ = 23.805, *p* < 0.001; Tukey post hoc *p* < 0.001) but not Context B (Tukey post hoc *p* > 0.05). There was a trend towards males having a greater number of active lever presses in Context A compared to females (Figure 3D,G; Context × Sex interaction *F*_1,18_ = 4.096, *p* = 0.058; Tukey post hoc *p* < 0.05). Mice pressed the active lever more in Context A versus Context B (Figure 3D,G; main effect of Context *F*_1,18_ = 59.626, *p* < 0.001). Overall, varenicline reduced the number of active lever presses compared to vehicle controls (main effect of Drug treatment *F*_1,18_ = 14.087, *p* = 0.001). To account for individual differences in lever pressing, the percentage change from baseline was calculated. This also revealed that varenicline reduced active lever responding compared to vehicle in Context A (Figure 3D,G; Context × Drug treatment interaction *F*_1,18_ = 10.086, *p* = 0.005; Tukey post hoc *p* < 0.01) but not Context B (Tukey post hoc *p* > 0.05). There was no effect of varenicline on the number of inactive lever presses (Figure 3D,G; all interactions *p* > 0.05).

## DISCUSSION

Models of alcohol‐seeking following punishment‐imposed or voluntary abstinence represent a translational procedure where abstinence is not manipulated by the experimenter. However, experiments examining the cross‐species use of this model and the translational value it provides have been lacking. This model has traditionally been used in rats. Here we showed that alcohol‐seeking following punishment‐imposed abstinence is possible in mice and that male mice demonstrated greater reinstatement of alcohol‐seeking behavior compared to female mice. We also examined the predictive validity of the model using naltrexone and varenicline, medications approved for use in humans and that have shown promise for reducing cravings in people with alcohol use disorder. When controlling for baseline responding, both naltrexone and varenicline reduced alcohol‐seeking behavior in mice. Together, these data support the use of the punishment‐imposed abstinence model in mice and highlight the validity of using this model to identify new, targeted treatments for cue‐ and context‐induced reinstatement.

### Alcohol‐seeking following punishment‐imposed abstinence in mice

One of the most prominent findings of our study was our observation of context‐dependent alcohol‐seeking behavior following punishment‐imposed abstinence in male and female mice. We were able to replicate findings from rats where mice reinstated alcohol‐seeking behavior in Context A, the alcohol‐associated context, but not Context B, the punishment context (Campbell et al., 2019, 2020; Marchant et al., 2013, 2016). Interestingly, female mice showed reduced active lever presses during the alcohol‐seeking test compared to males, which is comparable to our rat studies using a similar model (Campbell et al., 2021). Similar sex differences have also been observed using other substances of abuse and types of punishment. For example, research examining cocaine‐seeking following histamine punishment showed that female rats took longer to return to baseline operant responding compared to male rats (Holtz et al., 2013). One interpretation of the sex differences observed in seeking behavior following punishment includes sex‐dependent responses to aversive events, although we did not detect sex differences in shock sensitivity in the current study. Overall, the exact reasons for the sex differences in relapse‐like behavior in the current study are not clear, but it does implicate sex as an important variable to consider for treatment.

### The predictive validity of alcohol‐seeking following punishment‐imposed abstinence

Naltrexone is a nonselective opioid receptor antagonist with affinity for μ, δ, and κ receptors. It is approved for use in people with alcohol use disorder and has had broad success as an anti‐craving medication (Campbell et al., 2018; Maisel et al., 2013). However, human research on the effectiveness of naltrexone for treating alcohol use disorder in males and females independently has had mixed results, with studies often combining male and female treatment data to increase effect sizes (Baros et al., 2008; Garbutt et al., 2005; Greenfield et al., 2010). Here, we showed that when we accounted for baseline active lever responding, naltrexone reduced alcohol‐seeking behavior in both male and female mice. This aligns with several previous studies that have shown reduced cue‐induced reinstatement of alcohol‐seeking following naltrexone administration (Brown et al., 2023; Dayas et al., 2007; Le et al., 1999). Additionally, the same dose, pretreatment time, and route of administration (1 mg/kg, s.c., 15 min pretreatment) as that of the current study has been shown to reduce cue‐ and context‐induced reinstatement of alcohol‐seeking in male rats (Ciccocioppo et al., 2002; Marinelli et al., 2007). However, it is important to note that some studies on the effects of naltrexone on alcohol‐seeking have had mixed results, with Heidbreder et al. (2007) demonstrating no effect of naltrexone on alcohol‐seeking in male mice. This highlights that perhaps individual differences in prior alcohol consumption may influence responsiveness to naltrexone treatment. Indeed, recent research has shown that compulsive mice demonstrate greater responsiveness to naltrexone compared to low‐drinking mice; however, this was observed during alcohol consumption and not during seeking behavior (Brown et al., 2023). We did not examine the effect of naltrexone on alcohol consumption in the current study.

Varenicline is a partial agonist for α4β2 nicotinic acetylcholine receptors. Varenicline is FDA‐approved for smoking cessation and has been shown to reduce craving for alcohol in both smokers and nonsmokers (Litten et al., 2013; McKee et al., 2009; O'Malley et al., 2018). Here, we showed that varenicline reduced alcohol‐seeking in both male and female mice. This aligns with previous research where varenicline has also been shown to reduce alcohol consumption in male rats and both male and female mice (Holgate et al., 2017; Kamens et al., 2018; Wang et al., 2020). One limitation of the current study is that we did not conduct a dose–response curve for either varenicline or naltrexone. These medications have been widely used in the preclinical literature and doses previously shown to be effective at reducing alcohol‐related behaviors were chosen. One methodological consideration with our study is that we administered both naltrexone and varenicline acutely, prior to the final alcohol‐seeking test. However, in humans, treatments for substance use disorders are often administered chronically over the abstinence period. Despite this, acute drug treatment prior to examining alcohol‐seeking behavior is still informative, and this method has shown some promise for antidepressant treatments (Harmer et al., 2003; Robinson, 2016).

### Sex differences in alcohol consumption, operant self‐administration, and the response to punishment in mice

The drinking‐in‐the‐dark data was somewhat varied for all three experiments (Figure S1). While both male and female mice consumed alcohol during this phase of experimentation, the day seemed to impact consumption levels, and the effect of sex on alcohol consumption was unclear. This was contrary to expectations, as typically, female mice consume more alcohol in these binge drinking paradigms compared to male mice (Maddern, Letherby, et al., 2024; Mendez et al., 2024; Rivera‐Irizarry et al., 2023). To this end, we would like to highlight a potential limitation between our study and previous research. In our study, mice were group housed (2–5 mice per cage) to minimize disruption when transitioning from the drinking‐in‐the‐dark paradigm to operant conditioning. This meant that alcohol consumption was averaged for each mouse during this part of experimentation. Recent work has shown that group housing versus social isolation of male and female mice changes the microstructure of alcohol consumption patterns (Petersen et al., 2024). Thus, if the single housing of mice cannot be accommodated during this phase of experimentation, future research should consider tagging individual mice in a group housed setting to allow for accurate alcohol consumption measurements.

In the current study, we showed no sex differences in active lever responding for alcohol and high levels of alcohol consumption in Context A in both sexes. This demonstrates that mice consume alcohol in high quantities, equivalent to many frequently used binge drinking models (~1 g/kg in 30 min) (Rhodes et al., 2005). Additionally, we saw increased alcohol consumption in females compared to males in Context A. This is consistent with operant alcohol self‐administration paradigms in mice and our recent work in inbred alcohol‐preferring P rats (Blegen et al., 2018; Campbell et al., 2021; Sneddon et al., 2020, 2023). Whilst the reasons for consistent sex differences in alcohol consumption across these studies remain to be determined, we cannot rule out the influence of sex hormones or taste preference (Maddern, Walker, et al., 2024).

Our findings also highlight sex differences in Context B alcohol consumption, but this appears to be driven by Day 1 of Context B, where no foot shock is present. This aligns with the sex differences we observed in alcohol consumption during Context A self‐administration. Previous research has shown that female rodents with a history of alcohol use are more punishment‐resistant compared to male rodents, and these studies have used both foot shock punishment and quinine (bitter tastant) adulteration of alcohol (Maddern, Walker, et al., 2024; Sneddon et al., 2022, 2023; Sutton et al., 2021; Toivainen et al., 2024; Xie et al., 2019). While we did not observe sex differences in lever pressing when punishment was delivered (i.e., Phase 3: days 2 and 3), previous research has highlighted that sex differences in punishment‐resistant alcohol use are highly influenced by the context and timing of punishment delivery, along with stress, pain, and the rewarding properties of alcohol (Campbell et al., 2021; Maddern, Walker, et al., 2024; Radke et al., 2021; Toivainen et al., 2024). Finally, we did observe sex differences in inactive lever responding in Context B, where female mice reduced inactive lever responding across punishment day (despite the inactive lever having no programmed consequence) whereas male mice showed no change in inactive lever responding in Context B. This global reduction in lever responding in Context B in female mice may be due to a generalized stress response during punishment; however, we observed no sex differences in foot shock sensitivity.

One topic worth considering is the literature surrounding individual differences in the response to punishment. Indeed, previous research using quinine adulteration of alcohol has shown that high and compulsive alcohol drinking rodents show greater resistance to punishment compared to low alcohol drinking rodents (Augier et al., 2018; Brown et al., 2023; Siciliano et al., 2019). In the current study, it is likely that we were underpowered to examine individual differences in the response to punishment; however, individual differences in punishment suppression ratio can be observed in Figure S3. However, it has been shown that individual differences in the response to foot shock punishment only emerge when constant shock intensity is used, rather than increasing shock intensity over subsequent days, as in the current study (Marchant et al., 2018). Regardless, we saw a negative correlation between alcohol consumption and suppression ratio, suggesting that greater alcohol consumption was associated with greater suppression of operant responding when a foot shock was present.

## CONCLUSIONS

In conclusion, we demonstrate alcohol‐seeking following punishment‐imposed abstinence in a mouse model. We showed that both naltrexone and varenicline reduced alcohol‐seeking behavior in male and female mice, supporting the predictive validity of the model. This model could be used to identify more targeted treatments for relapse prevention in alcohol use disorder, and for future studies on neurobiological mechanisms of relapse.

## AUTHOR CONTRIBUTIONS

EJC designed the study. EJC, LT, and MK conducted all experiments. All authors conducted analyses and wrote the manuscript.

## FUNDING INFORMATION

This work was supported by an Australian Research Council Discovery Early Career Researcher Award (DE230100401) to EJC.

## CONFLICT OF INTEREST STATEMENT

All authors report no conflict of interest.

## Supporting information


Figures S1–S3

## Data Availability

All data are available upon request to the corresponding author.
