# Supplementary material for: Examining the predictive validity of alcohol‐seeking following punishment‐imposed abstinence in mice
Source: Alcohol Clin Exp Res (Hoboken). 2025 May 27;49(6):1337–50. doi: 10.1111/acer.70057 (PMC12174496; doi:10.1111/acer.70057)
Supplement: Supplementary file 1 — Figures S1–S3 [file ACER-49-1337-s001.pdf]

SUPPLEMENTARY FIGURES

EXAMINING THE PREDICTIVE VALIDITY OF ALCOHOL-SEEKING FOLLOWING PUNISHMENT-IMPOSED ABSTINENCE IN MICE

Linh Tran[1,2], Maria Kuznetsova[1,2], Elizabeth E. Manning[1,2] & Erin J. Campbell[1,2*]

[1]School of Biomedical Sciences and Pharmacy, Faculty of Health and Medicine, University of Newcastle, Callaghan, NSW, Australia
[2]Brain Neuromodulation Research Program, Hunter Medical Research Institute, New Lambton Heights, NSW, Australia.

Corresponding Author:

* Erin J. Campbell, PhD.
School of Biomedical Sciences and Pharmacy
Faculty of Health, Medicine and Wellbeing
The University of Newcastle
Callaghan, NSW, 2308, Australia
Email: erin.j.campbell@newcastle.edu.au

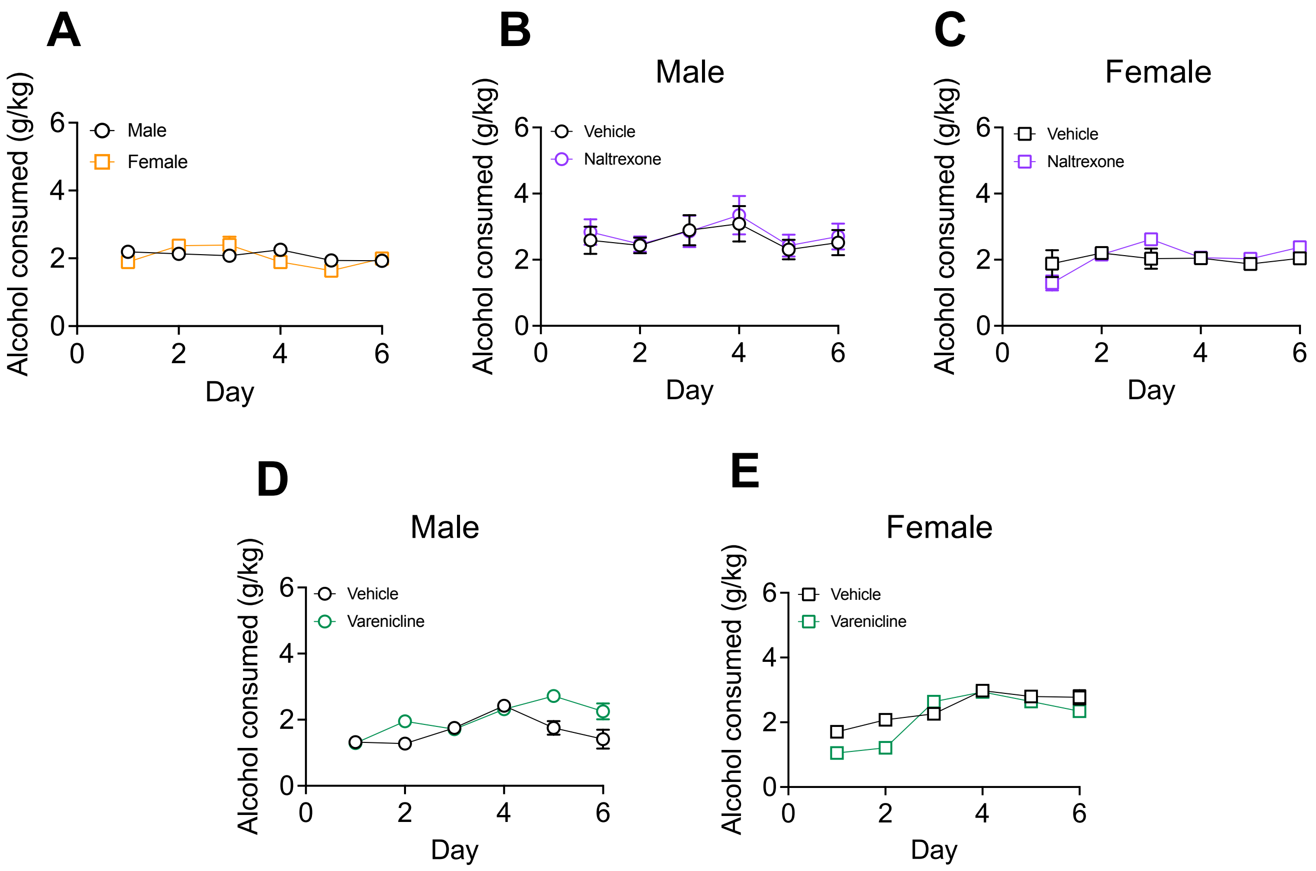

Figure S1. Phase 1: Drinking-in-the-dark data for all experiments. Both male and female mice consumed moderate to high amounts of alcohol during group housed drinking sessions. **A.** Experiment 1, Phase 1 alcohol consumption. **B.** Experiment 2a, Phase 1 male alcohol consumption. **C.** Experiment 2a, Phase 1 female alcohol consumption. **D.** Experiment 2b, Phase 1 male alcohol consumption. **E.** Experiment 2b, Phase 1 female alcohol consumption. Data presented as mean ± standard error mean.

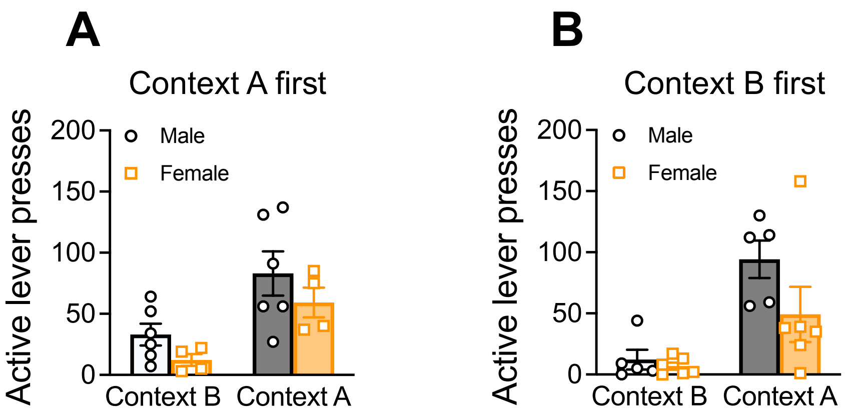

Figure S2. Effect of order of context counterbalancing on alcohol-seeking behaviour for Experiment 1. During the alcohol-seeking test, mice were exposed to both Context A and Context B (within-subjects testing). The order of testing of the two contexts was counterbalanced. There was no effect of the order of test context on the number of active lever presses during the alcohol-seeking test. **A.** Alcohol-seeking active lever presses when mice were tested in Context A first. **B.** Alcohol-seeking active lever presses when mice were tested in Context B first. Data presented as mean ± standard error mean.

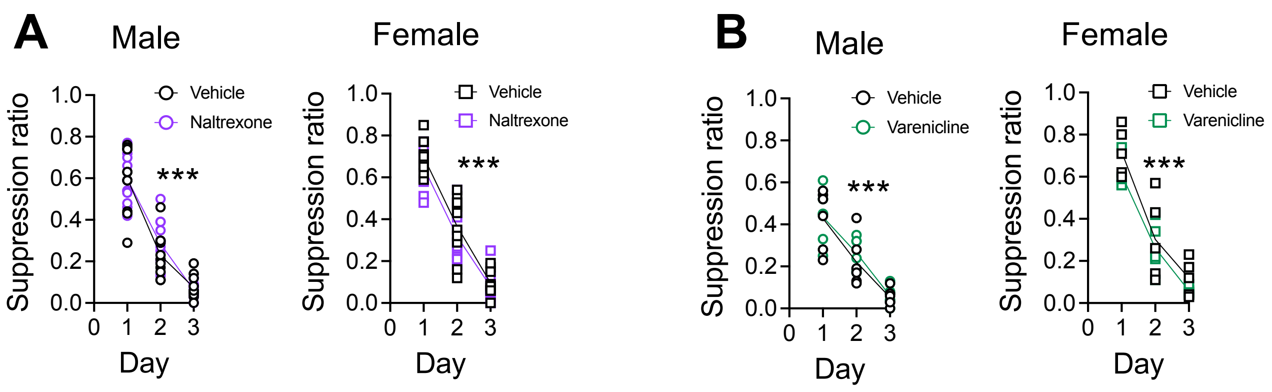

Figure S3. Punishment suppression ratio data for Experiments 2a and 2b. The suppression ratio was used to assess Context B punishment behaviour when accounting for baseline Context A alcohol self-administration. A suppression ratio of 0.5 reflects no change in the number of alcohol deliveries between Context A self-administration and Context B punishment. A suppression ratio < 0.5 represents a reduction in alcohol deliveries during punishment and a ratio > 0.5 represents an increase in alcohol deliveries during punishment. **A.** The suppression ratio reduced across day in both male and female mice for Experiment 2a. **B.** The suppression ratio also reduced across day in mice in Experiment 2b. Females had greater suppression ratios compared to males, but this was largely driven by punishment day 1 where no foot shock was present. Data presented as mean ± standard error mean. ***p<0.001, main effect of Day.
